# Supplementary material for: Tolerability of Atovaquone—Proguanil Application in Common Buzzard Nestlings
Source: Vet Sci. 2022 Jul 30;9(8):397. doi: 10.3390/vetsci9080397 (PMC9414624; doi:10.3390/vetsci9080397)
Supplement: Supplementary file 1 [file vetsci-09-00397-s001.zip › vetsci-1743233-supplementary.pdf]

Table S1. Mann-Whitney tests results.

Mann-Whitney tests results for comparisons of mean rank differences in blood chemistry parameters between "Pre-control" vs. "Pre-Mal" and "Post-control" vs. "Post-Mal" respectively. Adjusted p-values using Benjamini-Hochberg correction to account for multiple testing.

**Parameter**	**Group 1**	**Group 2**	**U-value**	***P*-value**	**adj. *P*-value**
ALB (g/L)	Pre-Control	Pre-Mal	157	0.217	0.537
	Post-Control	Post-Mal	33.5	0.081	0.396
AP (U/I)	Pre-Control	Pre-Mal	195	0.746	0.840
	Post-Control	Post-Mal	67.5	0.669	0.835
AST (U/I)	Pre-Control	Pre-Mal	206.5	0.962	0.962
	Post-Control	Post-Mal	55	0.743	0.835
BA (µmol/L)	Pre-Control	Pre-Mal	242	0.442	0.665
	Post-Control	Post-Mal	87	0.088	0.396
BuChE (KU/I)	Pre-Control	Pre-Mal	274	0.122	0.537
	Post-Control	Post-Mal	81.5	0.176	0.528
CK (U/I)	Pre-Control	Pre-Mal	241.5	0.443	0.665
	Post-Control	Post-Mal	73	0.438	0.789
GGT (U/I)	Pre-Control	Pre-Mal	257	0.238	0.537
	Post-Control	Post-Mal	68	0.636	0.835
LDH (U/I)	Pre-Control	Pre-Mal	224	0.728	0.840
	Post-Control	Post-Mal	75.5	0.341	0.767
TP (g/L)	Pre-Control	Pre-Mal	151	0.165	0.537
	Post-Control	Post-Mal	63.5	0.869	0.869

Table S2. Wilcoxon tests results.

Wilcoxon tests results for pairwise-comparisons of mean rank differences in blood chemistry parameters between "Pre-Mal" vs. "Post-Mal". Adjusted P-values using Benjamini-Hochberg correction to account for multiple testing.

**Parameter**	**U-value**	***P*-value**	**adj. *P*-value**
ALB (g/L)	43	0.413	0.465
AP (U/I)	6	**0.032**	0.097
AST (U/I)	45	0.320	0.465
BA (μmol/L)	48	0.206	0.371
BuChE (KU/I)	36	0.831	0.831
CK (U/I)	12	0.067	0.152
GGT (U/I)	5	**0.014**	0.064
LDH (U/I)	60	**0.014**	0.064
TP (g/L)	19	0.411	0.465

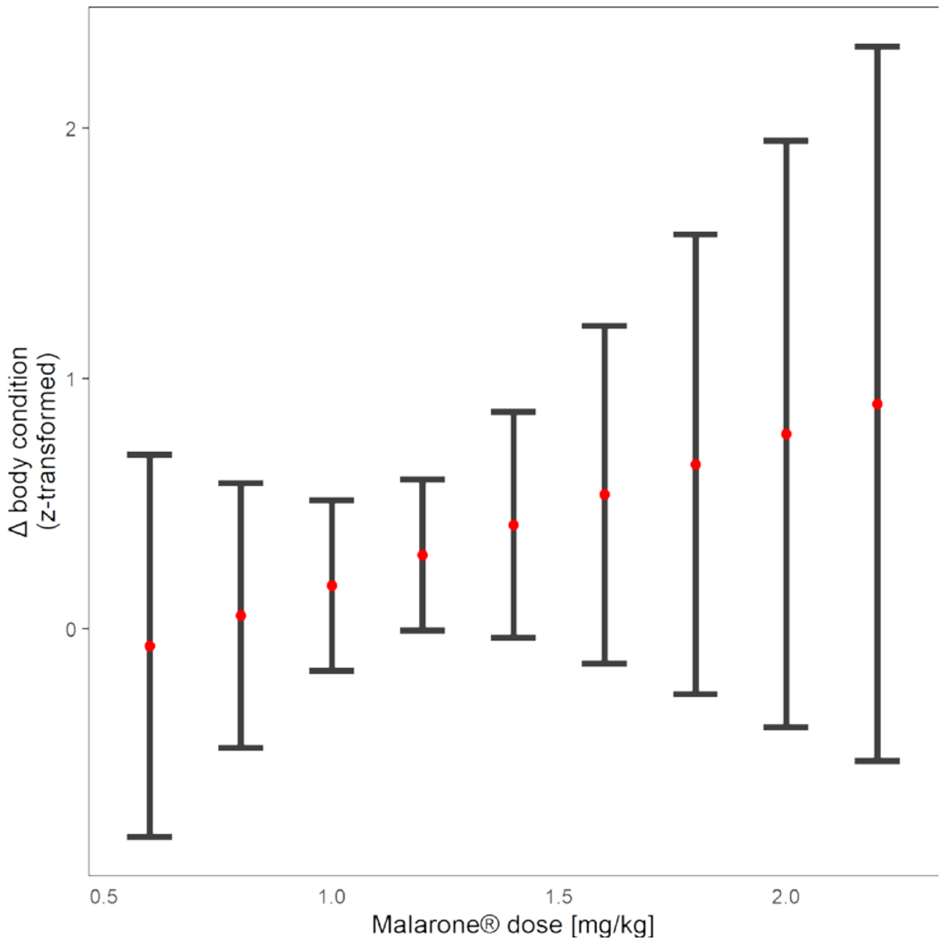

Figure S1. Predicted estimates for change in body condition (z-transformed) according to Malarone® dose (mg/kg) administered to common buzzard nestlings.

Change in body condition was calculated as the difference in body condition between first and second sampling. These are results from a linear mixed model testing the effect of drug dose on body condition (z-transformed). Red points are model estimates and black bars represent confidence intervals.

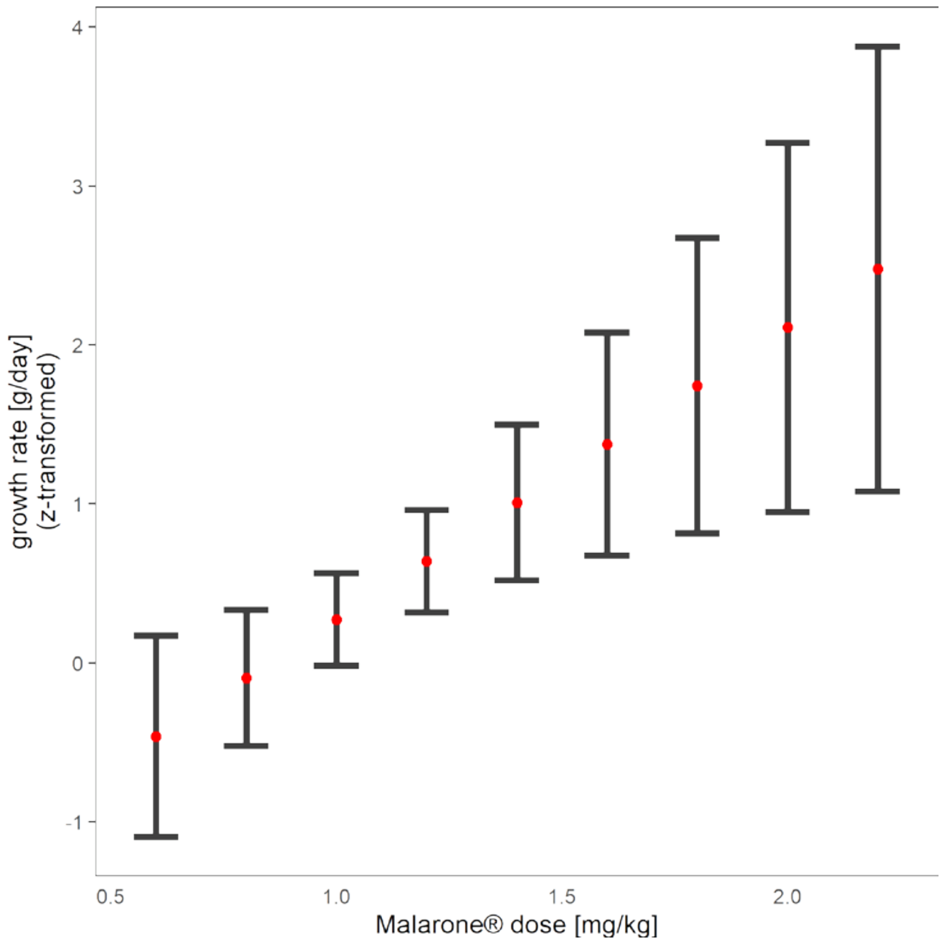

Figure S2. Predicted estimates for growth rate (g/day, z-transformed) according to Malarone® dose (mg/kg) administered to common buzzard nestlings.

These are results from a linear mixed model testing the effect of drug dose on body condition (z-transformed). Red points are model estimates and black bars represent confidence intervals.

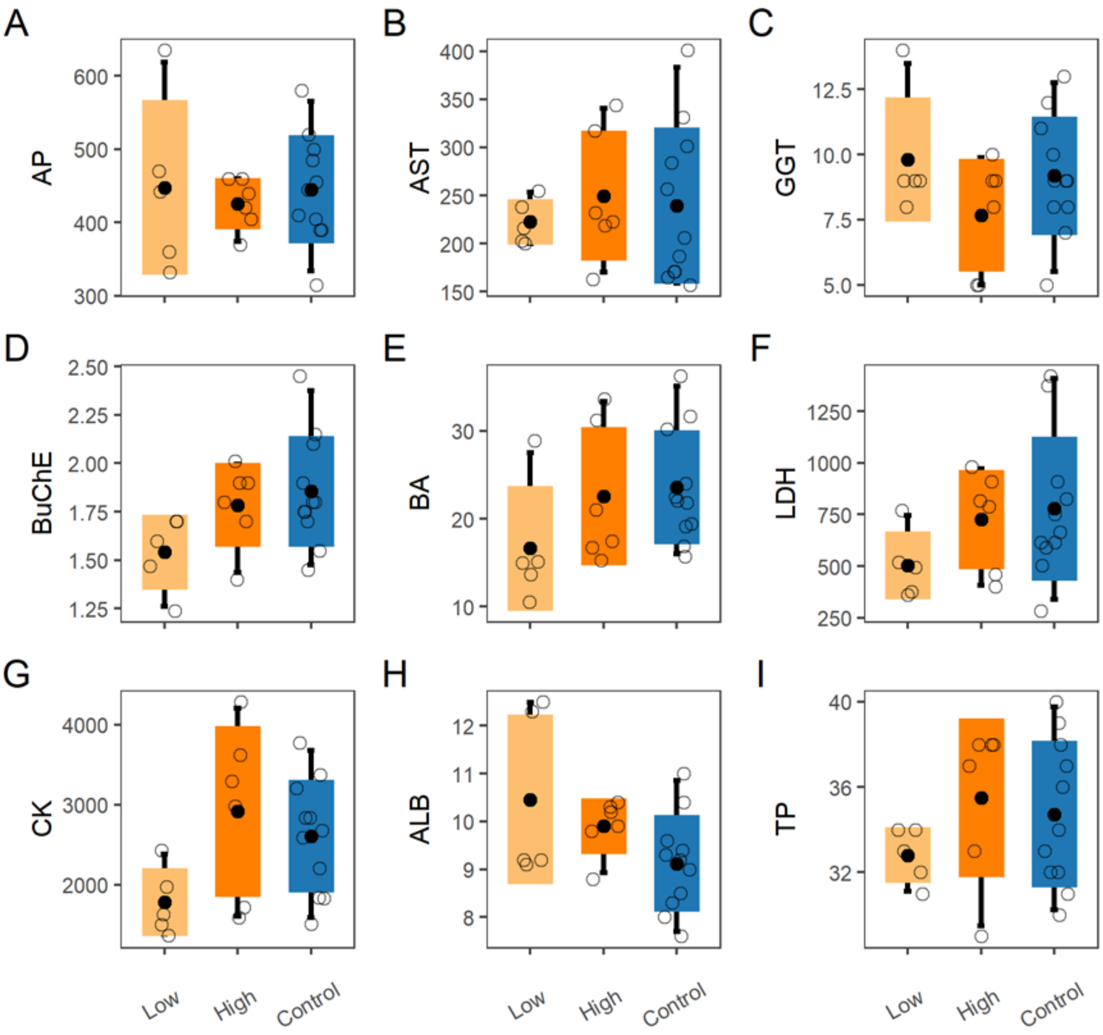

Figure S3. Observed value for nine blood parameters across nestlings grouped according to the amount of Malarone® dose administered (Low: < 9.68 mg/kg, High: > 9.68 mg/kl and Control: untreated).
No significant difference resulted from linear models testing the effect of dose groups on each nine blood parameters. Black points represent mean values and unfilled points are jittered observations. Boxplots are composed of a main bar, mean ± standard deviation and error bars as confidence intervals.
